# Cyclic neutropenia and concomitant IgA nephropathy: a case report

**DOI:** 10.1186/s12882-023-03179-1

**Published:** 2023-05-03

**Authors:** C. Kapogiannis, T. Zaggogianni, N. Stergiou, K. Kakleas, A. Kapogiannis, H. Gakiopoulou, C. Kanaka-Gantenbein

**Affiliations:** 1 Renal Unit, First Department of Paediatrics, National and Kapodistrian University of Athens, Aghia Sophia Children’s Hospital, Athens, Greece; 2 Renal Unit, Great Ormond Street Hospital, London, UK; 3 First Department of Paediatrics, National and Kapodistrian University of Athens, Aghia Sophia Children’s Hospital, Athens, Greece; 4 First Department of Pathology, Medical School, National and Kapodistrian University of Athens, Athens, Greece

**Keywords:** Case report, IgA nephropathy, Cyclic neutropenia, Angiotensin-converting-enzyme inhibitors, Corticosteroids

## Abstract

**Background:**

IgA nephropathy (IgAN) is universally recognized as one of the most common primary glomerular diseases in all ages. Cyclic neutropenia (CN) is a rare haematologic disorder that is associated with mutations of the ELANE gene. The co-occurrence of IgAN and CN is extremely rare. This is the first case report of a patient with IgAN and genetically confirmed CN.

**Case presentation:**

We report a case of a 10-year-old boy who presented with recurrent viral upper respiratory tract infections accompanied by several episodes of febrile neutropenia, haematuria, proteinuria and acute kidney injury. Upon first admission, his physical examination was unremarkable. His kidney function was impaired, whereas his urine microscopy showed evidence of macroscopic haematuria and proteinuria. Further workup showed elevated IgA. The renal histology was consistent with mesangial and endocapillary hypercellularity with mild crescentic lesions, while immunofluorescence microscopy showed IgA-positive staining, which was characteristic of IgAN. Moreover, genetic testing confirmed the clinical diagnosis of CN, therefore Granulocyte colony-stimulating factor (G-CSF) was initiated to stabilize the neutrophil count. Regarding proteinuria control, the patient was initially treated with an Angiotensin-converting-enzyme inhibitor for approximately 28 months. However, due to progressive proteinuria (> 1 g/24 h), Corticosteroids (CS) were added for a period of 6 months according to the revised 2021 KDIGO guidelines with favorable outcome.

**Conclusions:**

Patients with CN are more susceptible to recurrent viral infections, which can trigger IgAN attacks. In our case CS induced remarkable proteinuria remission. The use of G-CSF contributed to the resolution of severe neutropenic episodes, viral infections and concomitant AKI episodes, contributing to better prognosis of IgAN. Further studies are mandatory to determine whether there is a genetical predisposition for IgAN in children with CN.

## Background

IgA nephropathy (IgAN) is universally recognized as one of the most common primary glomerular diseases in all ages [1]. In European studies, it represents approximately 20% of glomerular diseases in childhood [2, 3]. It is characterized by IgA-1-containing immune deposits in the mesangial area of glomeruli. Genetic factors have also been identified to be involved in IgAN pathogenesis, especially at the level of IgA1 galactosylation [4–6]. However, the majority of paediatric patients with IgAN have a mild clinical course with less advanced histologic lesions [7–9]. IgAN often presents with macroscopic haematuria developing soon after or coinciding with an upper respiratory tract infection. Nevertheless, a small percentage of patients may present with nephritic syndrome or heavy proteinuria and sometimes with acute kidney injury (AKI), including crescentic disease [10]. Coppo et al. highlighted that 30–60% of paediatric subjects with IgAN will never face any decline in their glomerular filtration rate and have normal life expectancy, while approximately 10% will progress to end-stage renal disease (ESRD) within 10 years after diagnosis [2]. Remissions, either spontaneous or treatment-induced, are common according to Schima et al. [11]; therefore, the paediatric nephrologist often faces the dilemma, due to the unpredictable disease outcome, whether to treat paediatric patients with IgAN [2].

Cyclic neutropenia (CN) is a rare haematologic disorder with a prevalence of one in a million individuals in the general population, which is characterized by regular fluctuations in blood neutrophil count, leading to periodic neutropenia with a ~ 21-day periodic frequency [12, 13]. Recent genetic studies have shown that autosomal-dominant cyclic neutropenia is associated with mutations at locus 19p13.3 in the gene for neutrophil elastase (ELANE). The most common associated symptoms include fever, malaise, mucosal ulcerations, pharyngitis, cervical lymphadenopathy, and intermittent abdominal discomfort.

Secondary IgAN is uncommon. It has been reported in patients with various comorbidities, including gastrointestinal disorders, autoimmune conditions, various infections and neoplasms [14]. However, most of the molecular mechanisms responsible for glomerular deposition of IgG-IgA1 autoantibodies in patients with secondary IgAN have not yet been clearly elucidated. Moreover, according to Saha et al., some associations are coincidental in newly discovered IgAN [14].

To our knowledge, the co-occurrence of IgAN and CN in paediatric patients has been reported only twice until now [15, 16]. Specifically, the first case of a 5-year-old girl and the second case of a 10-year-old girl with CN and concomitant IgAN were published in 1978 and 2005 by Nash et al. and Matsukura et al. respectively. In both studies immunofluorescence showed strong granular staining for IgA to confirm the diagnosis of IgAN. However, CN was not genetically confirmed in either study [15, 16]. Moreover, prognosis was not discussed in abovementioned studies. Treatment strategy of IgAN was only described in Matsukura et al. study [16]. Of note, our study provides detailed information about the simultaneous occurrence of IgAN and genetically confirmed CN for the first time in the current literature, including data about diagnosis, prognosis, pathophysiology as well as treatment of both conditions. Similarities and differences between our study and aforementioned studies (Matsukura et al., Nash et al.) are depicted in detail in Table 1.

**Table 1:** Patient’s characteristics among different studies

Study	Gender	Age at presentation	Signs	Serum IgA	MEST-C score	Genetics (CN)	Treatment
**Current study**	Male	10 years	Macroscopic haematuria Proteinuria AKI	Elevated	M1E1S0T0-C0	Confirmed	ACE-inhibitor CS G-CSF
**Matsukura et al. (2005) **[16]	Girl	16 years	Haematuria Proteinuria	Elevated	NA	NA	CS G-CSF
**Nash et al. (1978) **[15]	Girl	5 years	Macroscopic haematuria Proteinuria AKI	Elevated	NA	NA	NA

### Clinical history and initial laboratory data

A 7-year-old boy was referred to our centre in June 2019 for investigation of his first episode of macroscopic haematuria, proteinuria, and kidney function impairment. A viral upper respiratory tract infection (tonsillitis) was reported seven days prior to the onset of the symptoms. From the age of 5 years, the patient has suffered several viral infections, mostly upper respiratory tract infections with concomitant cervical lymphadenopathy. Pregnancy and perinatal history were unremarkable. His family history was unremarkable, without reported renal disease or deafness.

Upon his first admission, our patient presented with a blood pressure of 115/56 mm/Hg. His vitals were otherwise within normal limits. Weight, height and body surface area were 22 kg, 1.22 m and 0.86 m^2^, respectively. Physical examination was unremarkable. Laboratory investigation showed elevated BUN at 67 mg/dl and creatinine at 1.21 mg/dl. His calculated glomerular filtration rate (GFR) using the “Bedside Schwartz” Eq. (2009) was 52 ml/min/1.73 m^2^. Blood gas was normal (pH: 7.4, pCO_2_: 35.7 mmHg, HCO_3_: 22.3 mmol/l). Urine dipstick revealed the presence of 3 + blood and 2 + protein; urine microscopy showed macroscopic haematuria and proteinuria. Twenty-four-hour urinary collection revealed non-nephrotic range proteinuria of 600 mg. Phase-contrast microscopy showed evidence of glomerular haematuria with more than 60% dysmorphic red blood corpuscles, whereas urine culture was sterile. The fractional sodium excretion (FeNa) was 0.9%. Further workup during his hospitalization showed elevated serum IgA 655 mg/dl (65–214 mg/dl) and IgG at 1960 mg/dl (812–1698 mg/dl), while IgM and IgG subclasses were within normal values for his age. Liver function was normal. Serological tests for hepatitis B, C and HIV were negative. Tests for anti-neutrophil cytoplasmic antibodies, anti-nuclear antibodies and anti-double-stranded DNA antibodies were negative. Laboratory investigation upon the patient’s first admission is shown in Tables 2 and 3. Renal ultrasound showed bilaterally increased echogenicity consistent with AKI, with the absence of calculi or signs of hydronephrosis.

**Table 2:** Initial laboratory investigation upon admission

Parameters	Value	References	Unit
Haemoglobin	110	120–145	g/dl
White blood cells	8 × 10^3^	8–11 × 10^3^	μl
Neutrophils	4.5 × 10^3^	1.5–7 × 10^3^	μl
Lymphocytes	3.5 × 10^3^	1.5–4 × 10^3^	μl
Monocytes	0.8 × 10^3^	0.1–1 × 10^3^	μl
Platelets	417 × 10^3^	150–440 × 10^3^	μl
C-reactive protein	30	< 10	mg/l
Erythrocyte sedimentation rate	100	< 20	mm/h
**Blood urea nitrogen**	**67**	15–54	mg/dl
**Creatinine**	**1.21**	0.3–0.6	mg/dl
Serum glutamic-oxaloacetic transaminase	24	10–60	U/I
Serum glutamic pyruvic transaminase	8	5–45	U/I
Gamma-glutamyl transferase	5	5–32	U/I
Total cholesterol	237	140–240	mg/dl
Triglycerides	196	45–170	mg/dl
Total protein	8.4	6.2–8.3	g/dl
Albumin	38	34–53	g/l
Sodium	137	134–152	mmol/l
Potassium	4.2	3.8–5.5	mmol/l
Phosphorus	5	3.7–5.5	mg/dl
Calcium	9	8.8–11.2	mg/dl
Magnesium	2	1.6–2.3	mg/dl
**Immunoglobulin A**	**655**	65–214	mg/dl
Complement component C3	160	90–180	mg/dl
Complement component C4	35	10–40	mg/dl
Antistreptolysin-O Titer	111	< 200	IU/ml
Antinuclear antibodies	(-)	1:40	-
Anti-double stranded DNA antibodies	(-)	< 200	IU/ml
Perinuclear anti-neutrophil cytoplasmic antibodies	(-)	-	u/ml
Antineutrophil cytoplasmic antibodies	(-)	-	u/ml

**Table 3:** Urine studies

**Dipstick urinalysis**	Value
Colour	Red
Ph	6.5
Specific Gravity	1015
Glucose	(-)
Blood	+ + +
Ketones	(-)
**Protein**	** + + **
Urobilinogen	(-)
Leukocyte esterase	(-)
Nitrite	(-)
**Urine microscopy**	Value
White blood cells	1–2 hpf
Red blood cells	> > 200 hpf
Squamous epithelial cells	none

### Diagnosis and management

**Fig. 1:**
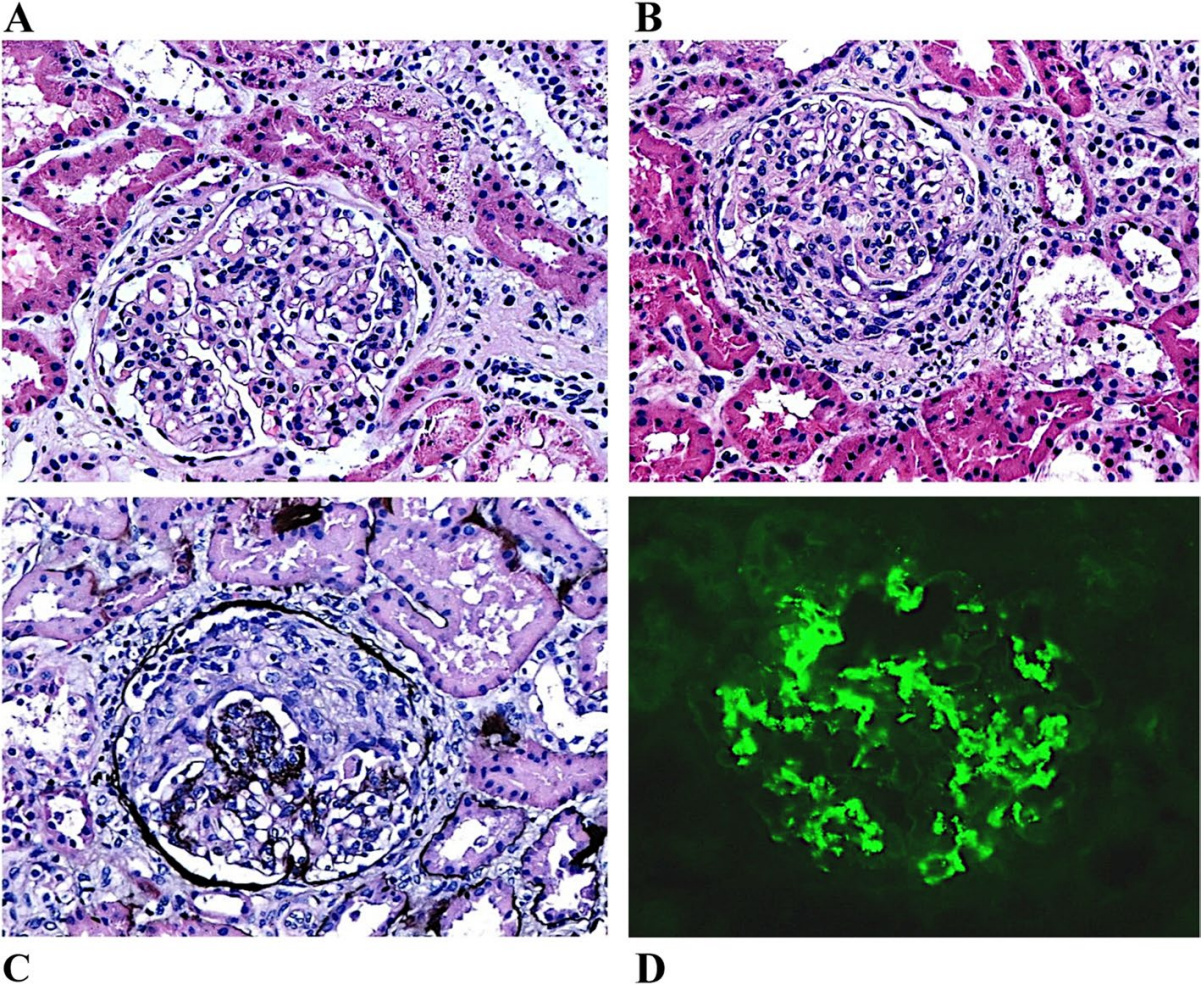
Typical pathological features of IgA nephropathy in renal biopsy. Light microscopy demonstrates glomerulus with increase of mesangial matrix and cellularity (HE × 400) (**A**), segmental endocapillary hypercellularity and cellular crescent (HE × 400) (**B**) and segmental endocapillary hypercellularity and cellular crescent (Silver × 400) (**C**) Immunofluorescence showing strong IgA (3 +) mesangial deposition (× 400) consistent with IgA nephropathy (**D**)

Subsequently, a left kidney percutaneous biopsy was performed as part of the investigation of AKI, haematuria and proteinuria. The tissue was examined by light and immunofluorescence microscopy. Twenty-nine glomeruli were found in the biopsy specimen, with only one of them (3.4%) being fibrotic. In the remainder, light microscopy revealed moderately increased mesangial matrix with mesangial (> 50%) and endocapillary (30%) hypercellularity (Fig. 1A, B). Moderate interstitial inflammation was also observed (lymphocytic infiltration), with no remarkable tubular atrophy. Crescents were revealed in 5 glomeruli (17.2%) (Fig. 1C). Small- and medium-sized vessels showed mild inflammation mainly composed of lymphocytes. Immunofluorescence microscopy included 4 glomeruli in every section, which showed intense granular staining for IgA (3 +) and C3 (2 +) in the mesangium (Fig. 1D). Electron microscopy was not performed. Based on the abovementioned findings, the patient was diagnosed with IgAN. The MEST-C score was M1E1S0T0-C1. After confirmation of IgAN, an angiotensin-converting-enzyme inhibitor (ACE-I) was initiated according to the 2012 Kidney Disease Improving Global Outcomes (KDIGO) guidelines [17]. Specifically, Ramipril was administered at a dose of 2.5 mg/m^2^/24 h, leading initially to gradual improvement of his urinary protein loss within seven days.

The patient was discharged; nevertheless, within the following months, he represented with periodic episodes of febrile neutropenia and monocytosis accompanied by viral upper respiratory tract infections. Associated symptoms included macroscopic haematuria and proteinuria with or without AKI. Physical examination was usually unremarkable except for tonsillitis, oral ulcers and cervical lymphadenopathy. His blood pressure values ranged between the 25^th^ and 50^th^ centile for his age and height. Laboratory investigation during these episodes revealed neutropenia, anaemia, increased inflammation markers, either microscopic or macroscopic haematuria, proteinuria and kidney function impairment. The patient during these episodes was treated symptomatically with IV hydration and management of biochemical and acid–base abnormalities. Broad spectrum antibiotics were administered in the presence of increased inflammation markers during severe neutropenia episodes. Interestingly, within five to six days after the initial presentation of each AKI episode, his kidney function was returning back to baseline range, without the use of immunosuppressive therapy.

Due to the fluctuation of his neutrophil count number and monocytosis, which followed a 21-day periodicity, genetic testing was performed, and the suspected clinical diagnosis of CN was confirmed. A heterozygous mutation (c.597 + 5G > A) was detected in exon 4 of the ELANE gene, classified as pathogenic according to the ACMG/AMP guidelines, in December 2019. Genetic testing was not performed in the patient’s parents and sister upon their refusal. Regarding the management of cyclic neutropenia, the patient was initially administered granulocyte colony stimulating factor (G-CSF) 2 mcg/kg/24 h in a five-day course before the expected episodes of neutropenia. Thereafter, the neutrophil count was periodically monitored. However, the patient continued to present episodes of severe febrile neutropenia with concomitant AKI; therefore, monthly administration of G-CSF was switched to alternate day (2 mcg/kg/24 h) with favorable outcome.

**Fig. 2:**
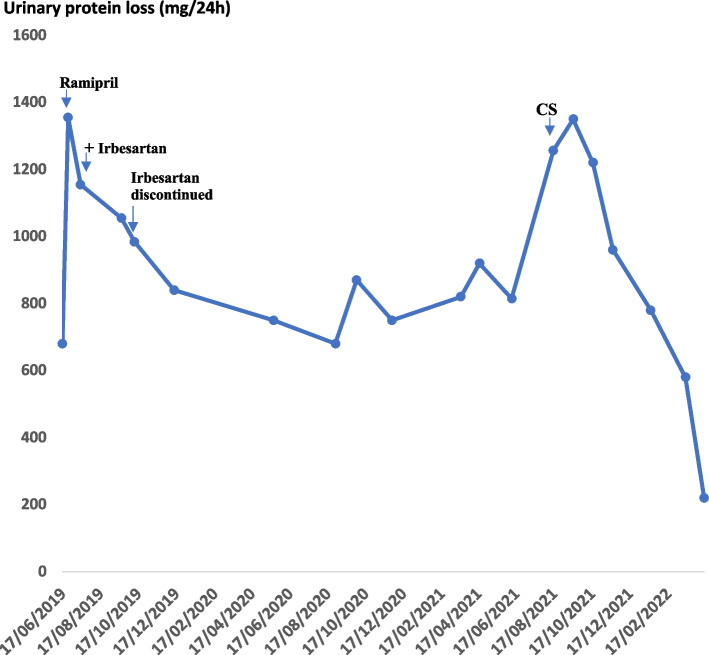
Urinary protein loss (mg/24 h) and treatment strategy according to the KDIGO guidelines from diagnosis of IgAN until 17/04/2022. Ramipril (2.5 mg/1.73m^2^/24 h) was administered initially with a good response. After 1 month of treatment the dose increased to 5 mg/1.73m^2^/24 h with further remission of proteinuria. Irbesartan was also started, but eventually discontinued soon due to low arterial blood pressure values (< 10^th^ for height and age). From 07’/2021 until 10’/2021 our patient presented with persistent proteinuria (> ~ 1 g/24 h), therefore CS were added resulting in significant remission of urinary protein loss

**Fig. 3:**
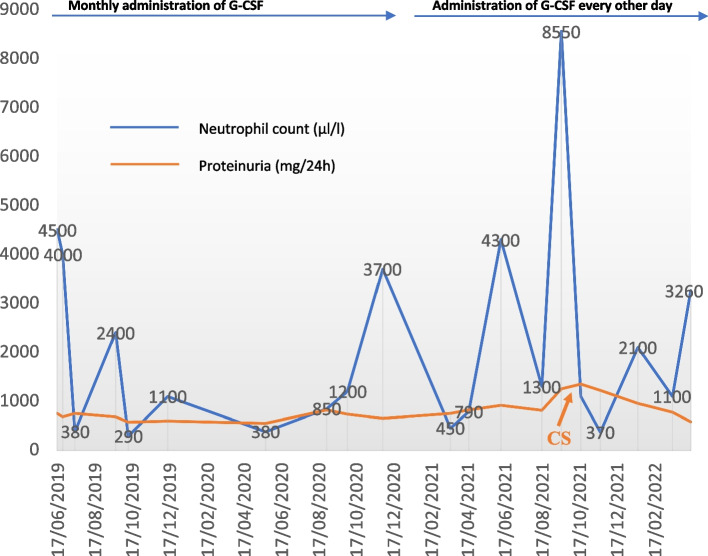
Combined G-CSF and Prednisolone effects towards neutropenia and proteinuria. G-CSF was administered monthly from diagnosis of CN until January 2021 and then switched to alternate day with remarkable control of the neutrophil count. Severe neutropenic episodes (neutrophils < 500μL), AKI events as well as hospitalizations were significantly reduced after administration of G-CSF every other day. Prednisolone was initiated in October 2021 with favorable outcome

However, in October 2021, the patient presented with another severe neutropenic episode accompanied by AKI (cr_max_: 3 mg/dl, BUN: 60 mg/dl). In addition, increased urinary protein loss (> 1 g/24 h) was persistently observed from July 2021 onwards. Therefore, it was decided to commence Prednisolone for 6 months according to the revised 2021 KDIGO guidelines [18]. The effect of CS in regard to proteinuria control from diagnosis of IgAN until July 2022 is shown in detail in Fig. 2. Figure 3 combines both G-CSF and Prednisolone effects towards neutrophil count, AKI episodes as well as proteinuria control respectively.

## Discussion

In this report, we describe in detail the rare case of a 10-year-old boy with genetically confirmed CN and concomitant IgAN. Our team included information about the renal biopsy findings, genetic investigation, treatment strategy as well as prognosis.

Regarding proteinuria treatment, our patient was treated with an ACE-I according to the updated 2021 KDIGO guidelines [18] after confirmation of IgAN showing a good response for a long period of time. However, because IgAN’s clinical presentation and outcome vary, it is indeed challenging for the clinician to promptly identify and decide whether to treat with CS or not subjects with IgAN who have the tendency to progress to ESRD [19]. Therefore, there is an ongoing debate worldwide about when to administer CS in paediatric patients with IgAN [19]. Of note, Coppo et al. (2019) was skeptical about the fact that the 2012 KDIGO guidelines considered proteinuria as the only risk factor to initiate CS in paediatric IgAN [2]. Nevertheless, the revised 2021 KDIGO guidelines did not add any additional risk factors regarding the initiation of immunosuppressive treatment in paediatric subjects with IgAN [18]. In our case, it was definitely a serious dilemma whether to initiate CS treatment or not even from the first AKI episode [2]. However, because of a relatively rapid improvement of our patient’s renal function during each AKI episode (within 7 days), effective proteinuria (< 1 g/24 h) and blood pressure control and considering long-term undesirable CS side effects [20], it was decided not to treat with CS for approximately 28 months after diagnosis. When proteinuria persisted above 1 g/24 h over a period of three months, it was decided to treat with prednisolone for 6 months, according to the revised 2021 KDIGO guidelines [18], at a dose of 2 mg/kg/24 h for 4 weeks, followed by alternate-day tapering for 5 months. After the initiation of CS, proteinuria gradually decreased, reaching remarkable remission after 2 months of treatment (Figs. 2 and 3). Of note, our patient’s kidney function and GFR have been within normal values after the administration of CS. In comparison to both previous studies, which reported the co-occurrence of IgAN and CN, the treatment strategy of IgAN was not described in the study by Nash et al., whereas Matsukura et al. reported alternate day of prednisolone administration at the time of diagnosis of IgAN. However, the duration of CS administration as well as the relevant guidelines used to guide treatment, were not included in abovementioned study.

Regarding long-term prognosis of IgAN, our patient’s risk of a 30% decline in estimated GFR or progressive renal disease 6.7 years after his diagnostic renal biopsy, according to the IgAN Paediatric Prediction Tool [21], was estimated at 10%. However, according to Trimarchi et al. more studies are required in the paediatric population to determine the role of this score in terms of individualized therapy selection [22]. Obviously, Nash et al. (1978) and Matsukura et al. (2005) did not include data in terms of IgAN prognosis [15, 16] in their studies, thus the Oxford classification of IgAN (MEST- score) was published in 2009, long time after both studies were published, by the International IgA Nephropathy Network and the Renal Pathology Society [22].

IgAN is the most prevalent primary glomerulonephritis worldwide, however its pathogenesis is multifactorial and indeed a focus of many IgAN studies. Multiple mechanisms are involved in its pathogenesis, including immunology, environmental factors as well as genetics. The most widely accepted “four hit” hypothesis implies that immunological mechanisms are involved in all aspects of IgAN development and play a crucial role. Of note, the precise pathogenetic mechanisms have not been fully determined yet. It is well known, that patients with IgAN produce an abnormal form of IgA1 [3], resulting in imbalanced increase of serum galactose-deficient IgA1. A variety of immune cells and molecules in innate and adaptive immunity are involved in the pathophysiology of IgAN. The formation of IgA-1-containing immune complexes results in mesangial deposition and complement activation inducing mesangial and endocapillary hypercellularity, segmental glomerulosclerosis and atrophying interstitial fibrosis [1, 2]. Interestingly, Matsukura et al. highlighted that chronic administration of G-CSF could have possibly induced the pathogenesis of IgAN causing glomerular injury [16], based on Cottle et al. study results, where risks of G-CSF treatment in patients with severe chronic neutropenia were highlighted [23]. However, after reviewing the current international literature about possible correlations between G-CSF administration and glomerular diseases, it became apparent that mainly case reports are described, without any clarification of the causal link between these two conditions [16, 24–26]. In our study, since the diagnosis of IgAN and CN was established almost simultaneously, Matsukura et al. hypothesis cannot be supported. Interestingly, Nash et al. study highlighted the formation of excess antibodies with suspected high avidity, caused by the recurrent viral infections and concomitant defective phagocytosis, as the possible inducing pathogenic mechanism of IgAN [15].

It is already known that viral infections can trigger IgAN attacks. Additionally, subjects with CN are prone to frequent viral infections, especially when developing severe neutropenia [15]; therefore, we believe that the persistent exogenous antigen stimulation may have caused the production of a continuous challenge to the mucosal immune system, leading to IgA overproduction. In addition, reduced antibody clearance due to defective phagocytosis [27], could have also contributed to the overproduction of pathogenic IgA1 immune complexes in our case. Of note, we observed that severely neutropenic episodes were often followed by viral infections and concomitant IgAN-attacks (Fig. 3). The relationship to infections as well as the finding of IgA-1-containing immune deposits in the mesangial area of glomeruli suggest immune complex formation as the pathogenic mechanism. However, we strongly believe that our patient has a concomitant genetical predisposition for IgAN. To our knowledge many studies, have recognized familial aggregation of IgAN [28–32] while large international, genome-wide association studies have reported several single nucleotide polymorphisms for IgAN [33, 34]. Many of them are found to be within or near to specific immune-related genes, including major histocompatibility loci. Thus, such genes may influence the progression of IgAN through interactions with environmental triggers (e.g., viral infections) [34].

Our patient presented several AKI episodes. Acute kidney injury is an uncommon presentation in children with IgAN. It mainly develops by two different pathogenic mechanisms. The first one includes an acute inflammatory process of the glomerular capillaries leading to proteinuria that induces proliferation of epithelial cells of Bowman’s capsule to form crescents. Notably, crescentic disease, is often accompanied by accelerated and irreversible loss of renal function [7, 10, 35]. According to Rajasekaran and Gutierrez et al. gross haematuria is the second cause of AKI in IgAN leading to reversible tubular damage or obstruction of the tubules by red-cell casts [36, 37]. Interestingly, our patient developed always reversible AKI episodes, especially when being severely neutropenic. However, knowing that our patient’s histopathology findings showed mild crescentic disease (M1E1S0T0-**C**1) upon admission, while on AKI, we believe that both mechanisms could have induced the development of AKI with a predominance of the second one. In summarize, we believe that viral infections in view of CN, triggered the overproduction of IgA1 immune complexes, which in turn resulted either in glomerular injury or gross haematuria-induced tubular dysfunction, leading to AKI. Interestingly, when G-CSF was switched to alternate day administration, severe neutropenic episodes as well as AKI episodes were significantly reduced (Fig. 3).

This study has limitation. Electron microscopy was not performed.

In conclusion, this is the first case report of a paediatric patient with IgAN and genetically confirmed CN. Our case demonstrates the intimate relationship between cyclic neutropenia, infections and AKI episodes associated with IgAN. This shows the importance of infections as a trigger and some evidence for the accumulations of immune complexes as the suspected pathophysiological mechanism. Of note, prednisolone administration led to instant remission of proteinuria and kidney function preservation. In addition, it was interestingly observed that alternate-day administration of G-CSF diminished the frequency of severe neutropenic episodes and subsequently AKI episodes and hospitalizations, thus improving quality of life. However, taking into account the complexity of IgAN, future large-scale randomized control studies are mandatory to elucidate whether there is indeed a genetic predisposition contributing to the pathogenesis of IgAN in children with CN. Undoubtedly, the use of individualized prognostic biomarkers to monitor disease activity as well as new treatment agents still remains a tempting approach for the future.

## Data Availability

Data sharing is available upon request.

## Abbreviations

IgAN: IgA nephropathy
CN: Cyclic neutropenia
AKI: Acute kidney injury
GFR: Glomerular filtration rate
ESRD: End-stage renal disease
FeNa: Fractional sodium excretion
KDIGO: Kidney Disease Improving Global Outcomes
ACE-I: Angiotensin-converting-enzyme inhibitor
G-CSF: Granulocyte colony-stimulating factor
